# Health impacts of expanding the health workforce across cadres under an incremental budget in Malawi

**DOI:** 10.1136/bmjgh-2025-022167

**Published:** 2026-08-04

**Authors:** Bingling She, Sakshi Mohan, Rachel E Murray-Waston, Sangeeta Bhatia, Martin Chalkley, Tim Colbourn, Joseph H Collins, Emilia Connolly, Eva Janoušková, Dominic Nkhoma, Paul Revill, Asif U Tamuri, Pakwanja Desiree Twea, Tara D Mangal, Joseph Mfutso-Bengo, Timothy B Hallett, Margherita Molaro

**Affiliations:** 1 Medical Research Council Centre for Global Infectious Disease Analysis, Jameel Institute, School of Public Health, Imperial College London, London, UK; 2 Centre for Health Economics, University of York, York, UK; 3 Institute for Global Health, University College London, London, UK; 4 Global Business School for Health, University College London, London, UK; 5 Ministry of Health, Government of Malawi, Lilongwe, Malawi; 6 Kamuzu University of Health Sciences, Blantyre, Malawi; 7 Centre for Advanced Research Computing, University College London, London, UK

**Keywords:** Health Personnel, Health policies and all other topics, Decision Making, Africa South of the Sahara

## Abstract

Like many others, Malawi’s healthcare system faces significant health workforce shortages largely due to budget constraints that limit training, recruitment and retention of staff. A crucial question is how to best allocate a limited incremental budget to expand different healthcare workers (HCW) cadres so that the potential health gains are maximised, which is more important now than ever considering recent withdrawal and reduction in donor funding. This research aims to provide a practical answer to this question. We designed a range of budget allocation scenarios for HCW expansion across cadres and used the ‘all diseases—whole healthcare system’ *Thanzi La Onse* (TLO) model to estimate the resulting population health outcomes. We find that, indeed, how to allocate the incremental budget among cadres is an important determinant of the potential health impact. Concentrating all of the budget on expanding a single cadre—such as clinical, pharmacy or nursing and midwifery—is not the most effective use of the resources, even when that cadre currently faces the greatest staffing shortages. Similarly, allocating the budget in a manner that mirrors the current distribution of spending and results in a uniform expansion across cadres does not generate the greatest possible gains. Instead, an allocation that uplifts staffing for multiple cadres, accounting for the additional time and costs required to meet the future healthcare needs, yields the greatest benefits. We conclude that, in the context of complex interplay between demography, epidemiology, treatment scope and effectiveness and health resource constraints, human resources for health (HRH) bottlenecks in achieving health gains are multifactorial and a needs-based balanced mix of cadres and skills is required for future HRH expansion. As such, health system models such as the TLO that capture this interplay can make potential contributions to strengthening HRH planning.

**WHAT IS ALREADY KNOWN ON THIS TOPIC**

- To date, very few studies have quantitatively analysed the potential future health impact of human resources for health (HRH) expansion in the context of evolving healthcare needs for a whole population. One existing study shows that investing in HRH expansion has the potential to achieve better health outcomes for Malawians, assuming a uniform expansion of multiple cadres.

**WHAT THIS STUDY ADDS**

- Using a detailed individual-based simulation model capturing the wide range of healthcare needs and the interdependency between cadres for delivering care, we have shown that HRH bottlenecks are multifactorial in driving future health outcomes and health gains could be achieved by allocating incremental resources to expand cadres in a carefully balanced way.

**HOW THIS STUDY MIGHT AFFECT RESEARCH, PRACTICE OR POLICY**

- This study answers the hard question of how to use an incremental budget for HRH expansion to achieve the greatest health gains in Malawi and demonstrates the essential use of system-wide modelling to support decision-making in complex health systems.

## Introduction

 Health systems in Malawi and many other low- and middle-income countries are challenged with shortages of human resources for health (HRH) and underfunding, in conjunction with increasing healthcare needs from growing populations.^1–7^ In such resource-constrained settings, the decision to expand HRH requires careful consideration of how limited funds are allocated among healthcare worker (HCW) cadres. This is a critical but hard question to answer in practice, considering the evolving healthcare needs and health burdens in a complex healthcare system that is undergoing demographic, epidemiological and economic changes, a detailed representation of the cadres required for delivering that care, and an understanding of the consequences that follow if the care is received or if it is not.

Whereas many studies have estimated the magnitude of HCW shortages based on recorded, targeted or simulated service levels,^1 2 4 8–12^ these analyses have not yet examined the resulting health consequences or sought to identify the optimal resource allocation for HRH expansion across cadres. While a linear constrained optimisation method has been used to investigate the marginal health impacts of investing in individual health worker cadres in Uganda and Malawi, static constraints and an epidemiological profile were assumed.^3 13^ Cost-effectiveness analyses of improving health workforce staffing levels and the patient outcomes and quality of care have been conducted; however, they were restricted to nurse cadres and mostly focused on high-income countries.^14^ Crucially, these analyses were not attached to epidemiological models, limiting their ability to estimate the health consequences of alternative resource allocation strategies accounting for interdependent epidemiology and service delivery dynamics. System dynamics modelling has also been used to inform health workforce planning. In this approach, macro-level health system behaviours, such as changes or movements of resources/services over time, are incorporated and the health workforce needs and health outcomes are normally driven by the changes of healthcare services that are strategically designed.^15 16^ Nonetheless, the micro-level interactions within or between health system resources for delivering care and patients needing care cannot be considered, making it difficult to explore how detailed changes of HCW cadres would affect health service provision and population health outcomes that are dependent on such interactions.

In contrast, the *Thanzi La Onse* (TLO) model,^17–19^ an individual-based simulation model for the Malawi healthcare system, captures demographic and epidemiological changes and interactions between individuals and the healthcare system, including links between HRH staffing levels, referral pathways and the incidence of comorbidities within the population. Therefore, this framework provides a new means to examine the health consequences of HRH expansion that can address existing gaps. Using this model, we have already quantified the potential health gains that could result from a uniform expansion of HCW cadres.^20^ This study now explores, based on the current staffing level, how incremental resources could be allocated across cadres for future expansions to maximise health gains in a complex, resource-constrained system.

## Methods

### The TLO model

The TLO model is an individual-based simulation model of healthcare service delivery in the public sector in Malawi. It models the healthcare needs of the population and how healthcare system resources, including HRH, consumables, facilities and equipment, are used to meet these needs and simulates health service provision and health burden in terms of disability-adjusted life years (DALYs) of the population. Capabilities of HRH (ie, total working time for delivering patient-facing/centred services) and consumables (ie, the current probability of availability of drugs and medical supplies), health system function in terms of diagnostic accuracy, referral practice and HCW competence, healthcare-seeking behaviour and service prioritisation policy are modelled constraints or parameters that impact the health service delivery and health outcome. Further details of this model and parameters can be found elsewhere.^17 20–23^

### HRH expansion scenarios

We model the HRH expansion in the TLO model by allocating an incremental budget among HCW cadres every year over the period between 2025 and 2034 (inclusive) and examine the health impacts of different allocation scenarios. We consider five groups of cadres—Clinical (C), Nursing and Midwifery (NM), Pharmacy (P), Disease Control and Surveillance Assistant (DCSA) or Health Surveillance Assistant (D) and Other (O), which include dental health, laboratory, mental health, nutrition and radiography cadres. These groups include all main cadres in the current workforce (excluding administrative staff) in the Malawi healthcare system, as detailed in online supplemental appendix A.1.1. For this study, the Clinical group includes medical officers or specialists, clinical officers or technicians and medical assistants, whose primary role involves direct clinical assessment, diagnosis, prescribing and/or management of patients. The inclusion of these cadres and the grouping is consistent with cadre categories used in Malawi Health Sector Strategic Plan (HSSP III) for 2023–2030^8^ and Human Resources for Health Strategic Plan (HRH SP) 2018–2022.^1 24^

Following Molaro *et al*,^20^ we assume a yearly HRH budget growth rate R=4.2%, that is, the incremental budget for HRH expansion for a given year is 4.2% of the total HRH cost of the previous year. We also assume the incremental budget and HRH cost here refer to staffing salaries only.

Each budget allocation scenario specifies what proportion of that incremental budget goes towards funding each of the five cadres, considering the differing salaries across cadres (see salary details and the scenario illustration diagram and in online supplemental appendices A.1.1 and A.1.2). We have designed 33 allocation scenarios (as listed in table 1) that can be grouped as below.

1. The ‘current allocation’ scenario, where the incremental budget allocation matches the existing funding allocation across cadres (ie, the incremental budget allocation proportions represent the relative total salary costs across the cadres in 2024). This allocation results in a uniform HRH expansion with staffing increase rates that are equal to the HRH budget growth rate R for every cadre every year (see explanations in online supplemental appendix A.1.3).
2. The 31 ‘equal allocation’ scenarios, where the incremental budget is allocated equally among each non-empty subset of the five cadres (including subsets of single cadres and the subset of all five cadres).
3. The ‘gap allocation’ scenario, where the incremental budget is allocated proportionally to the estimated extra cost of each cadre that is expected to be needed to meet all services’ demand as simulated in the TLO model. See more details of preparing this scenario in table 1. This scenario incorporates epidemiological trends, projected population health needs and additional workforce needs, capturing the combined shortages across multiple cadres relative to future healthcare demand.

**Table 1:** The incremental budget allocation scenarios and resulting workforce increase rates

Scenario group	Scenario label	Incremental budget allocation proportions					Average yearly increase rates of workforce size				
		p_C_	p_D_	p_NM_	p_P_	p_O_	x_C_	x_D_	x_NM_	x_P_	x_O_
No allocation	no_allocation	0%	0%	0%	0%	0%	0%	0%	0%	0%	0%
Current allocation	current_allocation	22%	23%	45%	3%	7%	4%	4%	4%	4%	4%
Gap allocation	gap_allocation	43%	2%	37%	14%	4%	7%	0%	4%	14%	2%
Equal allocation	C	100%	0%	0%	0%	0%	13%	0%	0%	0%	0%
	C=D	50%	50%	0%	0%	0%	8%	8%	0%	0%	0%
	C=O	50%	0%	0%	0%	50%	8%	0%	0%	0%	17%
	C=D=O	33%	33%	0%	0%	33%	6%	6%	0%	0%	13%
	C=NM	50%	0%	50%	0%	0%	8%	0%	5%	0%	0%
	C=NM=D	33%	33%	33%	0%	0%	6%	6%	3%	0%	0%
	C=NM=O	33%	0%	33%	0%	33%	6%	0%	3%	0%	13%
	C=NM=D=O	25%	25%	25%	0%	25%	5%	4%	3%	0%	11%
	C=P	50%	0%	0%	50%	0%	8%	0%	0%	26%	0%
	C=P=D	33%	33%	0%	33%	0%	6%	6%	0%	22%	0%
	C=P=O	33%	0%	0%	33%	33%	6%	0%	0%	22%	13%
	C=P=D=O	25%	25%	0%	25%	25%	5%	4%	0%	19%	11%
	C=P=NM	33%	0%	33%	33%	0%	6%	0%	3%	22%	0%
	C=P=NM=D	25%	25%	25%	25%	0%	5%	4%	3%	19%	0%
	C=P=NM=O	25%	0%	25%	25%	25%	5%	0%	3%	19%	11%
	C=P=NM=D=O	20%	20%	20%	20%	20%	4%	4%	2%	17%	9%
	D	0%	100%	0%	0%	0%	0%	12%	0%	0%	0%
	O	0%	0%	0%	0%	100%	0%	0%	0%	0%	24%
	D=O	0%	50%	0%	0%	50%	0%	8%	0%	0%	17%
	NM	0%	0%	100%	0%	0%	0%	0%	8%	0%	0%
	NM=D	0%	50%	50%	0%	0%	0%	8%	5%	0%	0%
	NM=O	0%	0%	50%	0%	50%	0%	0%	5%	0%	17%
	NM=D=O	0%	33%	33%	0%	33%	0%	6%	3%	0%	13%
	P	0%	0%	0%	100%	0%	0%	0%	0%	35%	0%
	P=D	0%	50%	0%	50%	0%	0%	8%	0%	26%	0%
	P=O	0%	0%	0%	50%	50%	0%	0%	0%	26%	17%
	P=D=O	0%	33%	0%	33%	33%	0%	6%	0%	22%	13%
	P=NM	0%	0%	50%	50%	0%	0%	0%	5%	26%	0%
	P=NM=D	0%	33%	33%	33%	0%	0%	6%	3%	22%	0%
	P=NM=O	0%	0%	33%	33%	33%	0%	0%	3%	22%	13%
	P=NM=D=O	0%	25%	25%	25%	25%	0%	4%	3%	19%	11%

(1) Here each scenario is specified with the incremental budget allocation proportions p_cadre_ and the resulting average yearly increase rate of workforce size x_cadre_. The derivation of x_cadre_ from yearly increase rates that are dependent on the workforce size of each previous year, the incremental budget allocated, and the salary for each cadre is detailed in online supplemental appendix A.1.3. To illustrate the equal scenario labels, ‘C’ refers to all incremental budgets allocated to the Clinical cadre, and ‘C=D’ refers to the incremental budget equally allocated to the Clinical and Disease Control and Surveillance Assistant cadres.

(2) An additional scenario, ‘no allocation’, is added to the table, where the HRH budget growth rate R=0 and the allocation proportions are zero for all cadres, representing ‘no expansion’ of the current health workforce. This scenario is used for preparing the ‘gap allocation’ scenario and also helps to show the magnitude of health benefits that can be achieved by any HRH expansion in the 33 allocation scenarios as compared to no expansion at all.

(3) To prepare the ‘gap allocation’ scenario, we first run the Thanzi La Onse simulation under the ‘no allocation’ scenario; second, calculate the unprovided health services between 2025 and 2034 due to unavailable HRH capabilities, as the difference between health service demanded and health service delivered; third, calculate the extra time needed for each cadre to deliver the unprovided services, by multiplying the time requirement for each cadre to deliver each service type and the quantity of each unprovided service type; finally, calculate the extra cost of each cadre in need by multiplying the extra time needed and the unit cost of each staff in that cadre, which is the annual salary divided by the annual patient-facing time.

HRH, human resources for health.

Each scenario is simulated 10 times in the TLO model with an initial population size of 100 000 in 2010. The reported results are the mean and 95% CIs of the outputs of 10 runs, which are scaled to the true Malawian population size of 14.5 million in 2010. The HRH expansion process from 2025 to 2034 within the TLO simulation period from 2010 to 2034 is illustrated in online supplemental appendix A.1.2.

### Main analysis

First, we compare the performance of all scenarios in terms of health consequences, as measured by disability-adjusted life years (DALYs), and health service volumes, as measured by the number of treatments delivered, in each, making the comparison to the ‘current_allocation’ scenario. Total DALYs and health service volumes are divided into six health areas: reproductive, maternal, newborn and child health (RMNCH), HIV/AIDS, malaria, tuberculosis (TB), non-communicable diseases (NCDs) and transport injuries. The specific causes of death or injuries in each health area are listed in online supplemental appendix A.1.6, which is among the leading causes of mortality and disability in Malawi.^17 20 25 26^ In all result figures, the scenarios are ranked in the same ascending order of total DALYs.

Next, we conduct a two-step analysis to explore if there are better allocation strategies than those defined above. In step one, we build a linear regression model (M1) to approximate the relationship between total DALYs incurred between 2025 and 2034, denoted by y, and average yearly increase rates of cadres, denoted by x_C_, x_D_, x_NM_, x_P_, x_O_; use Ordinary Least Squares method and the data of simulation results of 33 scenarios (data size=330, with 10 simulations per scenario) to estimate the coefficients and test the prediction performance of the regression model via another 100 randomly generated allocation scenarios by comparing the predicted and simulated outcome (data size)=100, with 1 simulation per scenario). In step two, we do an optimisation analysis (M2) to find the allocation strategy that minimises the DALYs, as approximated by M1. Here the average yearly increase rate of each cadre is dependent on the HRH budget growth rate, the incremental budget allocation proportion, the total salary cost of staffing of that cadre in 2024, and the total salary cost of staffing of all cadres in 2024 (see full constraint details in online supplemental appendices A.1.3 and A.1.4). We then use this scenario in the TLO model in order to estimate the total DALYs and compare it to the others.


(1) $$(M1)\;y=\begin{array}{l} \beta_{constant}+\beta_{C}x_{C}+\beta_{D}x_{D}\;+\\ \beta_{NM}x_{NM}+\beta_{P}x_{P}+\beta_{O}x_{O} \end{array}$$

(M2) min y s.t. constraints on x_C_, x_D_, x_NM_, x_P_, X_O_

### Sensitivity analysis

The simulations in the main analysis assume a fixed level of HRH budget growth rate (ie, R=4.2%), perfect consumables availability (ie, consumables are always available on request) and default health system function (ie, diagnostic accuracy, referral practice and HCW competence are informed by available data). We further conduct sensitivity checks by changing the HRH budget growth rate to higher and lower levels (ie, R=5.8% and R=2.6%), setting default consumables availability (ie, current availability level as informed by available data) or setting maximal health system function (ie, perfect diagnostic accuracy, referral practice and HCW competence). See online supplemental appendix A.1.5 for full details of health system parameter assumptions and the resulting ‘gap_allocation’ scenarios for the analyses. Notice that the sensitivity checks focus on the relative performances of all 33 scenarios as included in table 1 and do not include conducting optimisation analysis.

### Data sources and availability

The data source for HRH staffing, patient-facing time and service time requirements is the Malawi HSSP III for 2023–2030 from the Ministry of Health, Government of Malawi.^2 8^ The data source for HRH annual salary is Staff Returns from the Ministry of Health, Government of Malawi.^22^ All other data needed to run the TLO model simulation are available at the TLO model website.^21^ Simulation outputs used to generate tables and illustrations are available at a public, open-access repository.^27^

## Results

### The health outcomes of alternative scenarios

**Figure 1:**
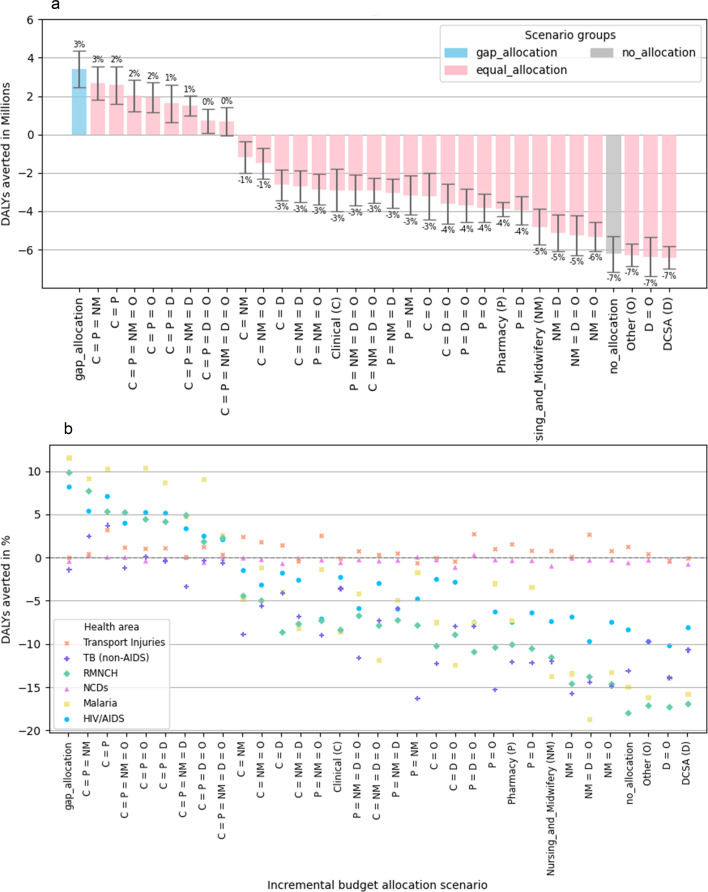
DALYs averted versus ‘current_allocation’ strategy, 2025–2034. (**a**) Total DALYs averted (annotated with percentage changes). (**b**) Relative DALYs by cause area averted. DALYs, disability-adjusted life years; DCSA, Disease Control and Surveillance Assistant; NCDs, non-communicable diseases; RMNCH, reproductive, maternal, newborn and child health; TB, tuberculosis.

Figure 1a shows the total DALYs averted in the period of 2025–2034 for each of the scenarios compared with the ‘current_allocation’ scenario (see full numerical results in online supplemental appendix A.2.2). The nine scenarios on the left that simultaneously expand Clinical, Pharmacy and/or Nursing and Midwifery cadres would achieve greater health gains than the ‘current_allocation’ scenario. The ‘gap_allocation’ scenario generates the greatest health gains and averts DALYs by 3.86% (95% CI 2.81% to 4.91%), equal to 3.41 (95% CI 2.46 to 4.36) million DALYs averted, compared with the ‘current allocation’ scenario. The next-most impactful scenarios are the ‘C=P=NM’ averting DALYs by 3.02% (95% CI 2.04% to 4.00%) and the ‘C=P’ scenario with a comparable performance; and the other six scenarios have per cent DALYs averted ranging between 0.75% and 2.28%.

By contrast, all other scenarios would have worse health outcomes than the ‘current_allocation’ scenario, with per cent DALYs averted ranging between −7.30% and −1.37% (where negative DALYs averted means more DALYs incurred). These include scenarios only expanding single cadres: for example, ‘Clinical (C)’−3.32% (95% CI −4.61% to −2.03%), ‘Pharmacy (P)’−4.43% (95% CI −4.89% to −3.96%), ‘Nursing and Midwifery (NM)’−5.49% (95% CI −6.58% to −4.40%).

Figure 1b shows the total DALYs averted split into health areas of RMNCH, HIV/AIDS, malaria, TB, NCDs and transport injuries (see online supplemental appendix A.2.1 for the figure with 95% CIs and online supplemental appendix A.2.2 for full numerical results). As compared to the ‘current_allocation’ scenario, the nine better scenarios on the left that simultaneously expand Clinical, Pharmacy and/or Nursing and Midwifery cadres achieve significantly better health gains in the areas of RMNCH, malaria and HIV/AIDS. Meanwhile, the other scenarios have worse health outcomes in the areas of RMNCH, malaria, HIV/AIDS and TB. The areas of NCDs and Transport injuries have no significantly different health outcomes across all scenarios, by contrast.

### The health services delivered in alternative scenarios

**Figure 2:**
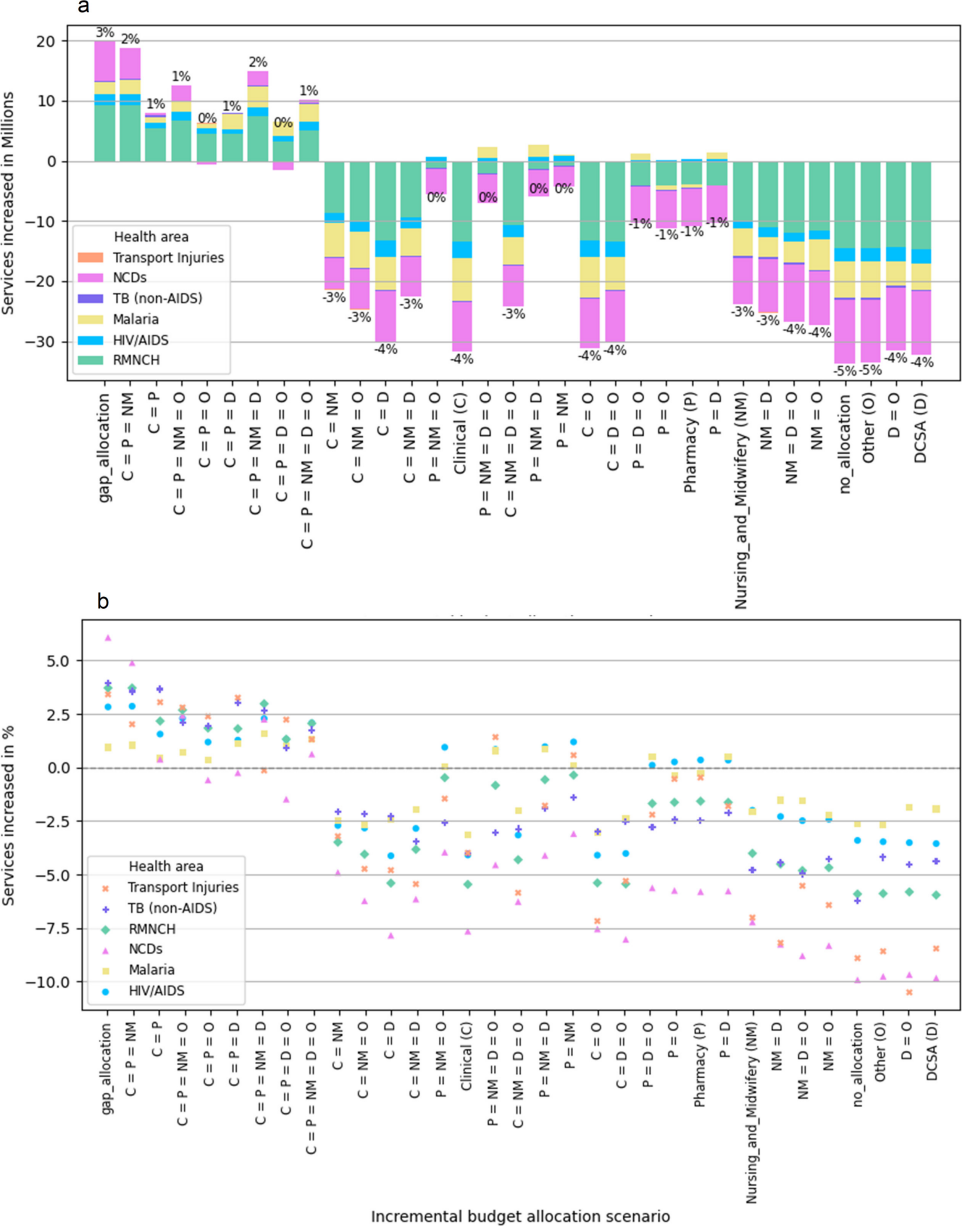
Increase in number of treatments by health area versus ‘current_allocation’ strategy, 2025–2034. (**a**) Absolute service increase (annotated with total percentage changes) and (**b**) relative service increase. DCSA, Disease Control and Surveillance Assistant; NCDs, non-communicable diseases; RMNCH, reproductive, maternal, newborn and child health; TB, tuberculosis.

Figure 2a,b show the absolute and relative increase of service volumes by health area across all scenarios as compared to the ‘current_allocation’ strategy (see the figure with 95% CIs in online supplemental appendix A.2.1 and full results in online supplemental appendix A.2.2). As expected, the overall patterns are consistent with those of the DALYs averted. The nine better allocation scenarios with lower DALYs generally have more services delivered, by approximately up to 2% for Malaria, up to 4% for RMNCH and TB, up to 3% for HIV/AIDS and transport injuries, and up to 6% for NCDs; among which, the ‘gap_allocation’ strategy would achieve the highest increases for these areas except malaria.

On the contrary, the worse scenarios with higher DALYs would have fewer services delivered overall than the ‘current_allocation’ scenario; among which, the worst scenarios with approximately 7% higher DALYs (ie, ‘Other (O)’, ‘DCSA (D)’, ‘D=O’) could be similar to ‘no expansion’, which itself would have services decreased approximately by 3% for HIV/AIDS and malaria, by 6% for RMNCH and TB, by 9% for transport injuries, and by 10% for NCDs. Nonetheless, scenarios that expand the Pharmacy cadre without the Clinical cadre (eg, ‘P=NM’) would have very modest decreases in total services and even significantly slight increases (<1%) for malaria and HIV/AIDS.

### Optimisation analysis

For step one, the estimated coefficients in regression model M1 are:


$$\begin{array}{l} \beta_{constant}=100.8007,\;\beta_{C}=-0.9464,\;\beta_{D}=-0.3928,\\ \beta_{NM}=-0.9712,\;\beta_{P}=-0.2536,\beta_{O}=-0.1851. \end{array}$$

The adjusted R-squared is 0.801. To interpret the coefficients, taking β_C_ as an example, it indicates that a 1% average yearly increase of Clinical cadre staffing between 2025 and 2034 corresponds to a decrease of 0.9464 million DALYs in total in this period.

The prediction performance is tested using 100 randomly generated allocation scenarios. We find that 96% of the random scenarios have simulated DALYs within the 95% prediction intervals. This shows good support for the regression model. More details can be found in online supplemental appendix A.2.3.

For step two, the ‘optimal solution’ to optimisation model M2 that is detailed in online supplemental appendix A.2.4 is:


$$\begin{array}{l} p_{C}=60.37\%,\;p_{D}=0\%\;,\;p_{NM}=9.22\%,\\ p_{P}=24.84\%\;,\;p_{O}=5.57\%, \end{array}$$

$$\begin{array}{l} (x_{C}=9.20\%,\;x_{D}=0,\;x_{NM}=0.99\%,\\ \;x_{P}=19.01\%,\;x_{O}=3.50\%). \end{array}$$

Running this scenario in TLO simulation shows that it would avert DALYs by 4.08% (95% CI 3.34% to 4.83%) as compared to the ‘current_allocation’, which is similar in magnitude to that achieved in the ‘gap_allocation’.

### Sensitivity analyses

As shown in online supplemental appendix A.3, the relative performances among the 33 scenarios of ‘current’, ‘equal’ and ‘gap’ allocations are overall robust by changing HRH budget growth rate, decreasing consumable availabilities and improving health system function. In particular, the ‘gap allocation’ and ‘C=P=NM’ scenarios remain the most impactful scenarios. Although limited consumable availability would hinder the full potential of health benefits of different HRH investment strategies, fewer DALYs would be averted as compared to the ‘current allocation’ scenario and improvement of health system function would further shift the impacts of investing in different cadres, as the Nursing and Midwifery cadre would have more impacts than the Pharmacy cadre across the scenarios.

## Discussion

We find that HRH bottlenecks in achieving health gains are multifactorial: concentrating all of the incremental budget on increasing capacity of a single cadre may yield limited benefit, whereas allocating the same budget to increase capacity of multiple cadres (specifically, Clinical, Nursing and Midwifery and Pharmacy) would produce much greater health gains. This indicates that the health impact of incremental HRH investment depends on how resources are allocated among cadres whose capacities are interdependent in enabling service delivery expansion, with substantial implications for the magnitude of achievable health gains.

The most impactful strategy identified was the ‘gap allocation’ strategy that aims to anticipate the future workforce needs across cadres and allocate resources accordingly. We also found that a linear regression model, estimated from the full set of simulated scenarios, can be used to derive an ‘optimal allocation’ of resources among cadres that would achieve highly comparable health benefits. Conversely, focusing expansion efforts on any single cadre alone may sacrifice health gains achievable through more diverse investment. For example, only expanding Clinical, Nursing and Midwifery, Pharmacy, DCSA or Other cadre would have approximately 3%–7% higher DALYs than uniformly expanding all cadres as in the ‘current allocation’ scenario, where the latter would be further outperformed by expanding multiple cadres based on estimated future needs as in the ‘gap allocation’ scenario. This does not imply that these individual cadres are unimportant, but rather that unbalanced workforce growth can undermine health system performance. However, the real-world impact of all strategies may differ from estimates in this analysis due to unmodelled implementation constraints such as shortages of staff accommodation and facility infrastructure.

Both the ‘gap allocation’ and ‘optimal allocation’ strategies result in substantially higher increase rates of Clinical and Pharmacy cadres compared with other cadres. This indicates greater shortages of these two cadres than others in meeting the increasing future health needs and avoiding potential health loss due to unmet care demand. This is consistent with the Malawi HSSP III that projects greater workforce needs for medical officers and pharmacy technicians than for nurses and midwives.^8^ It is also consistent with a 2017 Workload Indicators of Staffing Need (WISN) study in Malawi that indicates high shortages of these cadres based on their current workload.^10^ However, both HSSP III and WISN reports also suggest substantial needs for laboratory staff, a pattern not captured in this analysis due to the less well-specified roles of laboratory cadre within the TLO model. Nonetheless, our study has uniquely quantified the health consequences of alternative HRH expansion strategies and estimated the HRH needs dynamically and self-consistently, such that all consequences of constraints (ie, health resources, referral pathway, individual health outcome and future incidence of diseases) are jointly accounted for in a way that is not yet enabled in other tools. Furthermore, the finding that the simultaneous expansion of multiple cadres yields greater health gains than isolated expansion of any single cadre complements existing literature that primarily studies the impact of scaling up of a single cadre such as nurses and clinicians^3 13 14^: the real impact of expanding one cadre likely depends on concurrent changes in the availability of other cadres.

The relationship between capabilities of different HCW cadres and health outcomes is complex but was well represented by the linear model M1. This enabled us to develop a user-friendly tool (access here) for predicting health consequences (as measured in total DALYs) of any incremental budget allocation strategy for the purpose of supporting wider decision-making in HRH planning. In this tool, the user could input any proportion of the incremental budget allocated to each cadre (as long as they are non-negative and sum up to one), then it will translate the proportions into HRH increase rates and output the health consequences. This tool enables decision-makers to explore the approximate potential health outcome of any strategy of interest. However, reliability is more limited than the full execution of the TLO model simulation.

The results in areas of NCDs and transport injuries show that changes in HRH capabilities and health service volumes do not correspond with changes in health outcomes, unlike areas of RMNCH, malaria, HIV/AIDS and TB. These are consistent with findings in previous research, suggesting that current service scope and treatment effectiveness, as observed in the current Malawian health provision^17 28^ and as such captured in the TLO model, constrain the health gains achievable in these areas.^20^ Therefore, future research can explore interventions that aim to improve access to healthcare services and enhance treatment effectiveness, including training more specialist skills, which may improve health outcomes for both NCDs and transport injuries.^29–31^ In parallel, research can also explore interventions on risk reduction for NCDs, including poverty, harmful environment, poor diet, physical inactivity, tobacco use and harmful alcohol consumption, as these are established determinants of NCD burden,^29 32–35^ and interventions aimed at reducing traffic-related injuries, such as improving road infrastructure and strengthening the enforcement of traffic laws.^28 36^

Overall, this research has presented a simulation-based method to understand the complex relationships of HRH capability, health service provision and health outcomes and provided insights and tools that could inform relevant HRH policymaking. Nonetheless, there are several limitations.

Based on the existing staffing level and the fact of HRH shortages across cadres in Malawi,^1^ this analysis has focused on expanding HRH capabilities in the near future under an assumed incremental budget. The assumed HRH budget growth rate of 4.2% is consistent with the average annual HRH percentage increase between 2017 and 2024 in Malawi^37^ and the average annual percentage GDP growth between 1960 and 2020.^20^ However, this is an uncertain parameter considering HRH expenditure trends that account for potential declines in the overall health budget following recent donor funding cuts, as well as trends in other health spending.^20 38^ Besides, we did not incorporate workforce dynamics within the existing staffing, such as attrition or mobility, nor did we account for the costs associated with recruitment, training, supervision and retention for both existing and expanded staff. We also assumed a fixed salary for each cadre in the simulation period due to lack of empirical evidence of salary increases. A real-world HRH budget growth rate, the additional costs and potential salary increases can be incorporated in future analyses as data become available. Nonetheless, the sensitivity analysis of a lower growth rate of 2.6% shows that the central findings remain unchanged: the HRH bottlenecks in achieving health gains are multifactorial, and the needs-based simultaneous expansion of all cadres remains the best strategy.

We have assumed no task shifting among cadres, although such practices are implemented in African health systems to address workforce shortages and expand access to health services, particularly in HIV care and increasingly in the management of NCDs.^39–42^ The literature suggests that task shifting, by enabling more flexible use of available human resources, can improve access to services, strengthen continuity of care, maintain or enhance quality of care and reduce cost if implemented with sufficient training and management.^42 43^ Meanwhile, concerns remain regarding the adequacy of training, regulatory, supervision, management and sustainable financing, all affecting the quality and safety of care if not properly addressed.^44–46^ This analysis has made a simplified assumption to exclude task shifting. An important next step is to collect empirical evidence of the magnitude of task shifting in Malawi and examine the effects on health workforce expansion and population health. Besides, we have not considered potential changes in quality of care or HRH productivity brought by the expansion: the relationship between them remains an interesting research direction.

More flexibility of the HRH expansion process could be allowed, where the incremental budget allocation strategies can change every year or several years instead of being fixed throughout the period. Besides, the analysis is conducted at the national level: further analyses are required to inform policymaking on granular levels of the healthcare system, such as community, primary, secondary and tertiary levels or on district and regional levels.

Other health system parameters, such as healthcare-seeking behaviour and prioritisation policies of service provision, could also impact the health outcomes of HRH expansion strategies. In practice, we may expect care-seeking behaviour or service prioritisation to change in response to changes in HRH capabilities.

Finally, the impact of DCSA and other cadres, including laboratory (as mentioned above), mental and radiography staff, might be underestimated, as their roles are the least well specified in the TLO model.^2^ The Ministry of Health in Malawi is planning to increase staffing of these cadres: further calibration of the TLO model and exploration of the health impacts of investing in these cadres are needed to inform such decision-making.

## Conclusion

This study provides a model-based analytic framework for assessing the health consequences of alternative HRH expansion strategies under a fixed incremental budget. The TLO model of Malawi’s healthcare system uniquely captures demographic changes, epidemiological trends, constraints of health resources and treatment scope and effectiveness across multiple health areas, alongside the interactions between them. Using this model, we demonstrate that the HRH bottlenecks in achieving health gains are multifactorial in such a complex context. Strategically allocating incremental HRH investments across cadres, particularly Clinical, Nursing and Midwifery and Pharmacy, can yield substantially greater health gains than uniform or any single-cadre expansion. Strategies that anticipate future workforce needs can maximise health gains from limited budgets, whereas unbalanced allocation may result in significantly lower impact despite similar investment levels. To further inform real-world HRH policymaking, future research includes modelling task shifting among cadres and quantifying its impacts on service delivery and population health, as well as identifying optimal HRH investment strategies that support a balanced skills mix in both the current and future workforce.

## Supplementary material

10.1136/bmjgh-2025-022167 online supplemental file 1

10.1136/bmjgh-2025-022167 online supplemental file 2

## Data Availability

Data are available in a public, open-access repository. Data may be obtained from a third party and are not publicly available.
