# Supplementary material for: Health impacts of expanding the health workforce across cadres under an incremental budget in Malawi
Source: BMJ Glob Health. 2026 Aug 4;11(8):e022167. doi: 10.1136/bmjgh-2025-022167 (PMC13475466; doi:10.1136/bmjgh-2025-022167)
Supplement: Supplementary file 2 [file bmjgh-11-8-s002.docx]

### BMJ Global Health Author Reflexivity Statement

Adapted from Morton, B., Vercueil, A., Masekela, R., Heinz, E., Reimer, L., Saleh, S., Kalinga, C., Seekles, M., Biccard, B., Chakaya, J., Abimbola, S., Obasi, A. and Oriyo, N. (2022), Consensus statement on measures to promote equitable authorship in the publication of research from international partnerships. Anaesthesia, 77: 264-276. <https://doi.org/10.1111/anae.15597>

| **Study conceptualisation** | |
| --- | --- |
| 1. How does this study address local research and policy priorities? | Malawi’s health care system faces significant health workforce shortages largely due to budget constraints that limit training, recruitment and retention of staff. A crucial question is how to best allocate a limited incremental budget to expand different health care workers (HCW) cadres so that the potential health gains are maximised, which is more important now than ever considering recent withdrawal and reduction in donor funding. This study answers this question of how to use an incremental budget for HRH expansion to achieve greatest health gains in Malawi. |
| 2. How were local researchers involved in study design? | Via regular meetings and email communications, local researchers have provided health economics and health policy background information contributing to the contextualisation of the study. Local partners contributed to the validation of the modelling framework and ensured alignment with national health system priorities. |
| **Research management** | |
| 3. How has funding been used to support the local research team(s)? | Funding supported the involvement of Malawi-based collaborators in the contextualisation of the study and data interpretation. Funding also enabled collaboration with the Malawi health stakeholders and facilitated knowledge exchange and capacity strengthening in health systems modelling and evidence-based policy analysis. Local collaborators were actively involved throughout the research process and as co-authors, reflecting an equitable research partnership. |
| **Data acquisition and analysis** | |
| 4. How are research staff who conducted data collection acknowledged? | This study primarily used secondary administrative data from national and institutional sources. Individuals and collaborators who contributed to data provision, curation, and validation are recognised through co-authorship and formal citations where appropriate. |
| 5. How have members of the research partnership been provided with access to study data? | The study data is stored in public accessible online repositories, which are shared with members of the research partnership. |
| 6. How were data used to develop analytical skills within the partnership? | Data were used collaboratively within the partnership to develop and strengthen analytical skills, particularly in modelling of the health care system and interpretation of policy scenarios. Local collaborators were actively involved in data interpretation, model contextualisation, and validation, contributing their expertise to ensure accurate representation of the Malawi health system. This collaborative process facilitated knowledge exchange and supported capacity strengthening in quantitative analysis and evidence-based decision-making among partners. |
| **Data interpretation** | |
| 7. How have research partners collaborated in interpreting study data? | Research partners collaborated closely in interpreting study data through regular discussions and joint review of model assumptions, outputs, and policy scenarios. Local collaborators provided critical contextual expertise to ensure accurate representation of the Malawi health system and to interpret findings in relation to national priorities and implementation realities. This collaborative interpretation process ensured that conclusions were relevant, robust, and grounded in local health system knowledge. |
| **Drafting and revising for intellectual content** | |
| 8. How were research partners supported to develop writing skills? | Research partners were actively involved in manuscript development, including contributing to the discussions of findings and reviewing and revising drafts. Collaborative writing and feedback processes supported skill development in scientific writing and publication. This approach facilitated knowledge exchange and strengthened partners’ capacity to communicate research findings in peer-reviewed journals. |
| 9. How will research products be shared to address local needs? | Research findings will be shared with local health stakeholders, through reports, presentations, and annual Think Tank meetings in Malawi. Results will be disseminated through open-access publication to ensure accessibility. Where appropriate, modelling outputs and insights will be shared with collaborators to support ongoing policy analysis and capacity strengthening. |
| **Authorship** | |
| 10. How is the leadership, contribution and ownership of this work by LMIC researchers recognised within the authorship? | LMIC researchers were actively involved in the contextualisation and interpretation of the study, ensuring alignment with national priorities. Their leadership and intellectual contributions are recognised through co-authorship in accordance with ICMJE authorship criteria. |
| 11. How have early career researchers across the partnership been included within the authorship team? | Early career researchers across the partnership were actively involved in the research process, including study design, simulation running, data interpretation, and manuscript preparation. Their contributions are recognised through co-authorship in accordance with ICMJE authorship criteria. They were supported through collaborative analysis, mentorship, and participation in manuscript drafting and revision, promoting their professional development and research capacity. |
| 12. How has gender balance been addressed within the authorship? | Gender balance was actively considered in the composition of the authorship team. Contributions from researchers of different genders were recognised equitably, with authorship based on substantive intellectual contributions in accordance with ICMJE criteria. |
| **Training** | |
| 13. How has the project contributed to training of LMIC researchers? | The project contributed to the training of LMIC researchers through active involvement in contextualisation of study, data interpretation, and manuscript development. Collaborative engagement supported skill development in health systems modelling, quantitative analysis, and scientific writing. |
| **Infrastructure** | |
| 14. How has the project contributed to improvements in local infrastructure? | The project contributed to strengthening local research and analytical capacity through collaborative development and application of health systems modelling tools. Engagement with local partners supported the use of data-driven approaches for workforce planning and policy analysis. The partnership also strengthened institutional capacity (Kamuzu University of Health Sciences) by fostering ongoing collaboration, knowledge exchange, and the integration of analytical methods to support evidence-based decision-making within local health institutions. |
| **Governance** | |
| 15. What safeguarding procedures were used to protect local study participants and researchers? | This study used secondary data and did not involve direct interaction with human participants. Ethical approval was obtained from the College of Medicine Malawi Research Ethics Committee (COMREC, P.10/19/2820) in Malawi, and all data were handled in accordance with applicable data protection and confidentiality regulations. Collaborative engagement was conducted through professional and institutional channels, ensuring a respectful, inclusive, and safe research environment for all partners. |
